# Biochanin A Attenuates Ovariectomy-Induced Cognition Deficit *via* Antioxidant Effects in Female Rats

**DOI:** 10.3389/fphar.2021.603316

**Published:** 2021-03-16

**Authors:** Yanmeng Zhou, Bingbing Xu, Haiyang Yu, Wei Zhao, Xinxin Song, Yan Liu, Kainan Wang, Nikoli Peacher, Xiaomin Zhao, Han-Ting Zhang

**Affiliations:** ^1^ Institute of Pharmacology, Shandong First Medical University and Shandong Academy of Medical Sciences, Taian, China; ^2^ The Second Affiliated Hospital of Shandong First Medical University, Taian, China; ^3^ Departments of Neuroscience and Behavioral Medicine and Psychiatry, Rockefeller Neurosciences Institute, West Virginia University Health Sciences Center, Morgantown, WV, United States

**Keywords:** estrogen, biochanin A (BCA), memory, neuroprotection, postmenopause, ovariectomy, rat

## Abstract

**Background:** Impairment of memory and cognition is one of the major symptoms in women with postmenopausal disorders due to estrogen deficiency, which accounts for the much higher prevalence of Alzheimer’s disease in females. Biochanin A (BCA), a natural phytoestrogen, has been reported to protect neurons against ischemic brain injury. However, the neuroprotective effects of BCA in the postmenopausal-like model of ovariectomized (OVX) rats remain to be investigated.

**Methods:** All the rats except for the sham group underwent the resection of bilateral ovaries. Seven days after the OVX surgery, rats were randomly divided into six groups: sham, OVX, OVX + BCA (5 mg/kg), OVX + BCA (20 mg/kg), OVX + BCA (60 mg/kg), and OVX + estradiol (E2; 0.35 mg/kg), which were administrated daily by gavage for 12 weeks. Learning and memory were examined using the Morris water-maze test before the end of the experiment. Morphological changes of the rat hippocampus were observed by HE staining and electron microscopy. Malondialdehyde (MDA), superoxide dismutase (SOD), and glutathione peroxidase (GSH-Px) in the hippocampus were measured. The effect of BCA on cell viability was measured in the presence of hydrogen peroxide (H_2_O_2_) using CCK8. Flow cytometry was used to measure neuronal apoptosis and reactive oxygen species (ROS) induced by H_2_O_2_. Expression of Bcl-2, Bax, and Caspase-3 was determined by Western blotting using hippocampal tissues and primary cultures of hippocampal neurons.

**Results:** Chronic treatment with BCA mimicked the ability of E2 to reverse the deficit of learning and memory in the Morris water-maze test in OVX rats. BCA normalized OVX-induced morphological changes as revealed by HE staining and electron microscopy. In addition, BCA significantly decreased the levels of MDA, the biomarker of oxidative damage, and increased the activity of the intracellular antioxidant enzymes SOD and GSH-Px in OVX rats. Further, in primary cultures of hippocampal neurons, BCA reversed H_2_O_2_-induced decreases in cell viability and accumulation of ROS. Finally, BCA reversed OVX- or H_2_O_2_-induced increases in Bax and Caspase-3 and decreases in Bcl-2 in the hippocampus and primary cultures of hippocampal neurons.

**Conclusion:** These results suggest that BCA improves memory through its neuroprotective properties in the brain under the circumstance of estrogen deficiency and can be used for treatment of memory loss in postmenopausal women.

## Introduction

Gender is a strong contributor to the vulnerability to Alzheimer’s disease (AD), the most common neurodegenerative disease and the primary cause for dementia. Women have a higher prevalence of AD (Chapman et al., 2011). Approximately two-thirds of AD patients are females, in particular postmenopausal women, contributing to over 60% of all those affected (Brookmeyer et al., 2011). This suggests that deficiency of estrogens is a critical risk factor of AD in women under the postmenopausal condition. It has been shown that, in postmenopausal women, the concentrations of circulating ovarian hormone gradually decrease as age increases (Carroll et al., 2007). Hormone replacement therapy (HRT) has been successfully used for the management of menopausal symptoms. Epidemiological analyses have revealed that women receiving HRT in their perimenopausal period are at a lower risk of AD (Henderson and Brinton, 2010), while those untreated are more likely to suffer from AD (Rocca et al., 2008; Rocca et al., 2010). A growing number of studies suggests that ovarian hormones, including E2 and progesterone, play a neuroprotective role (Pike et al., 2009; Engler-Chiurazzi et al., 2016). However, large clinical trials have indicated that HRT causes serious side effects on the breast and uterus (Beral and Million Women Study Collaborators, 2003). Therefore, it is necessary to develop drugs exerting estrogenic properties with minimum adverse effects, to prevent and treat estrogen deficiency-related diseases.

Biochanin A (BCA), a major isoflavone constituent found in red clover, cabbage, chickpea, and some other herbal dietary supplements, exhibits a variety of pharmacological properties, including antihyperglycemic (Harini et al., 2012), antioxidative and anti-tumor (Mishra et al., 2008), anticholinergic (Biradar et al., 2014), and dopaminergic neuron protective effects (Chen et al., 2007). More importantly, BCA has estrogenic properties and is considered to have fewer side effects (Sklenickova et al., 2010). However, the neuroprotective property of BCA remains largely unknown, especially its contribution to the cognitive deficit developed in the postmenopausal women. Thus, it was of interest to know if BCA produced neuroprotective and memory-enhancing effects in ovariectomized (OVX) rats, a model of postmenopausal syndrome, which is highly related to oxidative stress (Zhou et al., 2016).

Oxidative damage has a critical contribution to cognitive declines in aging and neurodegenerative diseases (Facecchia et al., 2011; Michael et al., 2016), which is mediated by reactive oxygen species (ROS). Hydrogen peroxide (H_2_O_2_) has been extensively used as an inducer of oxidative stress models of oxidative damage and neuronal apoptosis (Liu et al., 2011). In the present study, we investigated the neuroprotective effects of BCA in the hippocampus of OVX rats *in vivo* and in primary cultures of hippocampal neurons treated by H_2_O_2_ *in vitro*.

## Materials and Methods

### Animals and Surgery

Sixty adult female Sprague Dawley rats at 6 months old, weighing 270–330 g (Jinan Pengyue Laboratory Animal Breeding Co. Ltd, Jinan, China) were used in the experiments. Animals were housed in plastic cages with controlled temperature (24 ± 2°C) and humidity (40–50%) and a 12-h light/dark cycle. The experimental protocols were conducted in accordance with the National Institute of Health Guide for the Care and Use of Laboratory Animals. Animal use was approved by the Committee of Animal Experimental Ethics of Shandong Frist Medical University, China. After a 7-days adaptation period, bilateral ovariectomy (OVX) or a sham operation was performed under anesthesia with pentobarbital sodium (30 mg/kg, i.p.). A longitudinal incision was made inferior to the rib cage on the dorsolateral body wall. In the OVX rats, the bilateral ovaries were exteriorized, ligated, and excised, whereas rats subjected to the sham surgical procedure had only a piece of fat excised. All the rats were intraperitoneally injected with penicillin (20,000 U/kg) for three days after the surgery to prevent infections.

### Drug Treatment and Body Wright Monitoring

Biochanin A (purity >98%) and β-estradiol (E2; purity >98%) were purchased from Sigma-Aldrich (Saint Louis, MO, United States).

Seven days after the surgery, rats were divided into six groups (n = 10 per group): sham, OVX, OVX + BCA (5 mg/kg), OVX + BCA (20 mg/kg), OVX + BCA (60 mg/kg), and OVX + E2 (0.35 mg/kg). BCA and estradiol were dissolved in 10% Tween 80 and 10% ethanol and administered by gavage every morning for 12 weeks. The doses of BCA and E2 and the treatment duration were determined based on previous studies (Aguiar et al., 2006; Biradar et al., 2014; Alauddin et al., 2018) and our preliminary experiment.

The body weights were monitored once a week for 4 weeks during the experiment.

### The Morris Water-Maze (MWM) Test

Learning and memory of rats were evaluated using the MWM test following the procedures as described previously (Zhang et al., 2008). In order to exclude the variations caused by the circadian rhythm, animals were trained and tested each day from 10:00 AM to 5:00 PM. In the navigation experiment, rats were trained for two sessions per day for four consecutive days, with the interval of 4 h between sessions. In each trial, the rat was allowed to locate the hidden platform by swimming within 120 s. If it failed to find the platform within 120 s, the rat was guided to the platform by the experimenter and allowed to stay for 10 s. The escape latency, which was defined as the time reaching the platform, was recorded with the cut-off time 120 s. On day 5, the platform was removed starting the probe trial test, during which the time spent in the target quadrant, where the platform had been placed, and the number of times crossing the target quadrant were recorded for 120 s.

### Hematoxylin and Eosin Staining (HE Staining)

On the second day after the MWM test, all the rats were anesthetized with pentobarbital sodium (30 mg/kg, i. p.) before removal tissues for further experiments. Two rats from each group were randomly selected for morphological observations. The left brains were stained with HE and the right brains were taken for electron microscopy. HE staining was performed as described previously (Gao et al., 2019). The hippocampus was dissected and immersed in 4% paraformaldehyde. The tissues were embedded in paraffin wax and cut into 5 μm slices. The sections were stained in hematoxylin solution for 5 min and then decolorized in 1% hydrochloric acid ethanol for 10 s. Brain sections were then stained with eosin for 2 min. The slides were observed under the light microscope (Olympus BH-Z, Japan).

### Electron Microscopy

The specimen preparation was performed as described previously (Gao et al., 2019) with minor modifications. The hippocampal tissues were immediately fixed in 2.5% glutaraldehyde and stored in the same solution at 4°C overnight. After fixation, samples were post-fixed in 1% osmium tetroxide for 2 h at room temperature. Fixed, dissected tissues were dehydrated in a graded alcohol series and embedded in Eponate 12 medium. After rinsing, fixation, dehydration, and epoxy resin embedding, ultrathin slices were loaded into a copper-loaded grid with ead-uranium double dye. After thorough cleaning and concentration, samples were placed under the perspective electron microscope (FEI-TECNAI-G20) for examining and capturing the ultrastructure changes of neurons.

### Measurement of Serum 17β-Estradiol Levels

All rats were deprived of food overnight before anesthesia. Blood samples were collected from the abdominal aorta, and serum was separated by centrifugation at 1,500 × g, 4°C for 10 min. Serum estradiol was assayed using enzyme-linked immunosorbent assay (ELISA) kits (Wuhan Elabscience Biotechnology Co., Ltd, Wuhan, China) according to the manufacturer’s instructions.

### Preparation of Hippocampal Tissues

This was performed as described previously (Guo et al., 2019). In brief, the hippocampi of eight rats of each group were collected and homogenized individually with external ice-cold saline bath. Then, the homogenates were centrifuged at 1,500 × g for 20 min at 4°C, and the supernatants were collected and stored at −80°C before assays.

### Detection of SOD, GSH-Px, and MDA

The protein content was determined using bicinchoninic acid protein assay kits (Beijing Solarbio Science andTechnology Co. Ltd, Beijing, China). The levels of SOD, GSH-Px, and MDA were detected following the manufacturer’s instructions (Nanjing Jiancheng Bioengineering Institute, Nanjing, China). The results were shown as nmol/mg protein.

### Primary Cultures of Hippocampal Neurons

Rat primary hippocampal neurons were obtained from newborn rat pups within 24 h and the procedures described previously (Banker and Cowan, 1977), with minor modifications. The hippocampus was dissected and washed with HBSS to remove the mixed blood vessels, and then cut into 1 mm^3^ pieces before digesting with 0.125% trypsin at 37°C for 15 min. Cells were resuspended in the DMEM medium supplemented with 10% horse serum (FBS, Sijiqing, Hangzhou, China) and left alone for 15 min, after which the upper layer of single-cell suspension was collected. Dissociated hippocampal cells were plated on polylysine-coated plates and 1 h later, DMEM was replaced by Neurobasal media (Gibco, Carlsbad, California, United States) supplemented with B27 (Gibco). Cultures were incubated for 7–10 days *in vitro* at 37°C in 5% CO_2_. A half of the media was replaced every 3 days. The cultured neurons were identified by MAP-2.

### Neuronal Viability

The viability of neurons was evaluated using the CCK8 assay (Beijing Tongren Institute of Chemistry, Beijing, China). Cells were seeded into 96-well plates at a concentration of 6 × 10^3^ cells/well. The CCK8 solution was added to the cells, which were then incubated for 1–4 h. The optical density values were detected using a spectrophotometer (Tecan, Swiss) at 450 nm.

Different concentrations of BCA (10^−7^–10^−3^ mol/L) or H_2_O_2_ (100, 200, 400, and 800 μmol/L) were added to primary hippocampal neurons of PLL-coated 96-well plates, each with eight multiple wells. After 24 h or 12 h culture**s**, 10 µL CCK8 reagent was added and the optical density value**s** were determined at the wavelength of 450 nm using the automatic microplate analyzer.

### Flow Cytometric Analysis of Apoptosis

Cell death was evaluated using an Annexin V-FITC apoptosis detection kit (KeyGEN, Nanjing, China). Briefly, neurons were transferred to 5 ml tubes containing Annexin V-FITC and incubated in the dark for 10 min. The neurons were then stained with Annexin V-FITC binding buffer and propidium iodide (PI) before incubating in the dark for 15 min. Apoptosis was quantified using flow cytometry (BD Biosciences, Franklin Lakes, NJ, United States). FACSDiva software version 5.0.2 (BD Biosciences, San Jose, CA, United States) was used for data acquisition and analysis.

### Measurement of ROS Levels

ROS levels were estimated following the procedures published previously (Best et al., 1999). The assay was based on deacetylation of 2′,7′-dichlorofuoresceine diacetate (DCFH-DA) following ROS-mediated oxidation, which further elicits a fluorescent product, i.e., DCF. Cells were cultured with 10 µM DCFH-DA (Beyotime, Shanghai, China) for 1 h before analyzing *via* flow cytometry by measuring DCF fluorescence at an excitation/emission wavelength of 488/525 nm, respectively. FACSDiva software version 5.0.2 was used to analyze fluorescence intensities and determine ROS production.

### Western Blotting

The stocked supernatants of hippocampal tissues of three rats in each group were used for Western blotting. Cells were washed by PBS and lyzed (Beyotime, Shanghai, China), followed by oscillating for 30 s and incubating on ice for 30 min. After determination of concentrations using BCA kits (Solarbio, Beijing, China), the proteins were separated on SDS-PAGE and transferred onto polyvinylidene difluoride membranes (GE Healthcare, Little Chalfont, United Kingdom). The membranes were incubated at 4°C with primary antibodies, including caspase-3 (1:1,000, Cell Signaling Technology), Bcl-2 (1:1,000, Santa Cruz Biotechnology), Bax (1:1,000, Santa Cruz Biotechnology), and β-actin (1:10,000, Cell Signaling Technology), and then probed by horseradish peroxidase-conjugated second antibodies (Zhongshan Golden Bridge, Beijing, China). The immunoreactivity was visualized using Enhanced Chemiluminescence (ECL) reagents (Amersham) and images acquired on AI-600 System (GE, United States).

### Data Analysis and Statistics

Statistical analyses were performed using GraphPad Prism 8.0 statistical software (GraphPad software, Inc.). Data were expressed as means ± standard error (SE). Data were analyzed using one-way analysis of variance (ANOVA) followed by *post hoc* Dunnett's tests to compare differences among multiple groups, with *p* < 0.05 considered statistically significant.

## Results

### BCA Prevented OVX-Induced Body Weight Gain and Increased Serum Estradiol Levels

**FIGURE 1:**
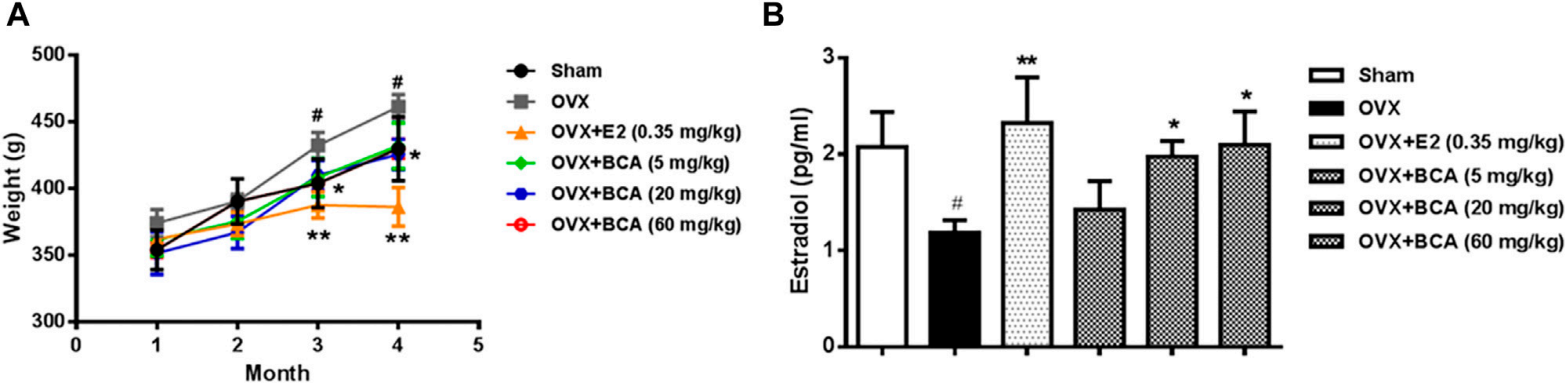
The effects of BCA on body weights and serum estradiol levels in the ovariectomized (OVX) rats. **(A)** Changes in body weights of sham and OVX rats treated with or without estradiol (E2) or BCA (5–60 mg/kg). **(B)** Changes in serum estradiol levels. Values shown are means ± S.E. ^#^ *p* < 0.05 vs. Sham; *p < 0.05, **p < 0.01 vs. OVX alone; n = 10.

The body weight of OVX rats was significantly greater than the sham controls (F_(5,54)_ = 5.38, *p* < 0.05). The OVX-induced body weight gain was suppressed by estradiol at 0.35 mg/kg (*p* < 0.01) and BCA at doses of 5–60 mg/kg (*p* < 0.05) in dose-dependent manner (Figure 1A). Compared to the sham controls, OVX rats showed significant decreases estradiol in serum (F_(5,54)_ = 4.89, *p* < 0.05; Figure 1B), suggesting a successful model of OVX. This was blocked by estradiol (*p* < 0.01); BCA mimicked the ability of estradiol to increase estrogen contents in OVX rats (*p* < 0.05; Figure 1B).

### BCA Improved Learning and Memory of OVX Rats

**FIGURE 2:**
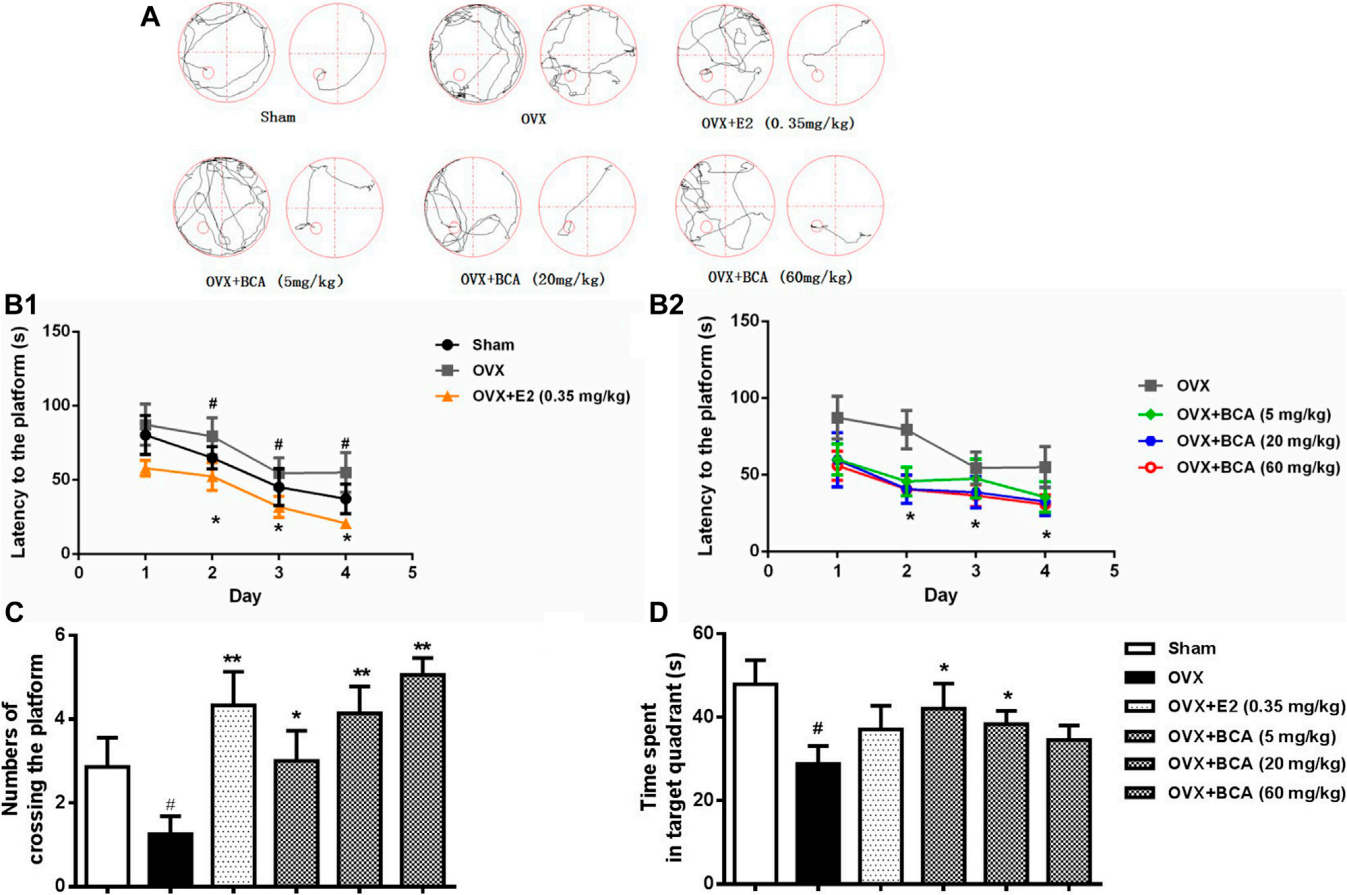
The effects of BCA on learning and memory of OVX rats in the Morris water-maze (MWM) test. **(A)** Representative swimming trajectory of rats during the acquisition training (left: day 1, right: day 4). **(B)** The escape latency of navigation during the 4-days acquisition training. **(C)** The number of crossings into the target quadrant in the probe trail test. **(D)** The time spent in the target quadrant in the probe trial test. Values shown are means ± S.E. ^#^ *p* < 0.05 vs. Sham; *p < 0.05, **p < 0.01 vs. OVX alone; n = 10.

In the MWM test, the rats displayed decreased escape latency during the acquisition training over time, indicating improved learning after repeated training. Compared to the sham, the OVX rats had longer escape latency (F _(5,54)_ = 3.95, *p* < 0.05), indicating spatial learning impairment induced by OVX (Figure 2A,B); this was attenuated by treatment with BCA and estradiol (*p* < 0.05). In the probe trial test, both the number of crossings and the time spent in the target quadrant were decreased in the OVX rats relative to the sham (F _(5,54)_ = 4.63, both *p* < 0.05; Figure 1C,D); these were blocked by BCA and estradiol (F _(5,54)_ = 5.54, *p* < 0.05 or *p* < 0.01), although it was noted that the significances varied in the time spent in the target quadrant.

### BCA Improved Morphology of Hippocampal Neurons in OVX Rats

**FIGURE 3:**
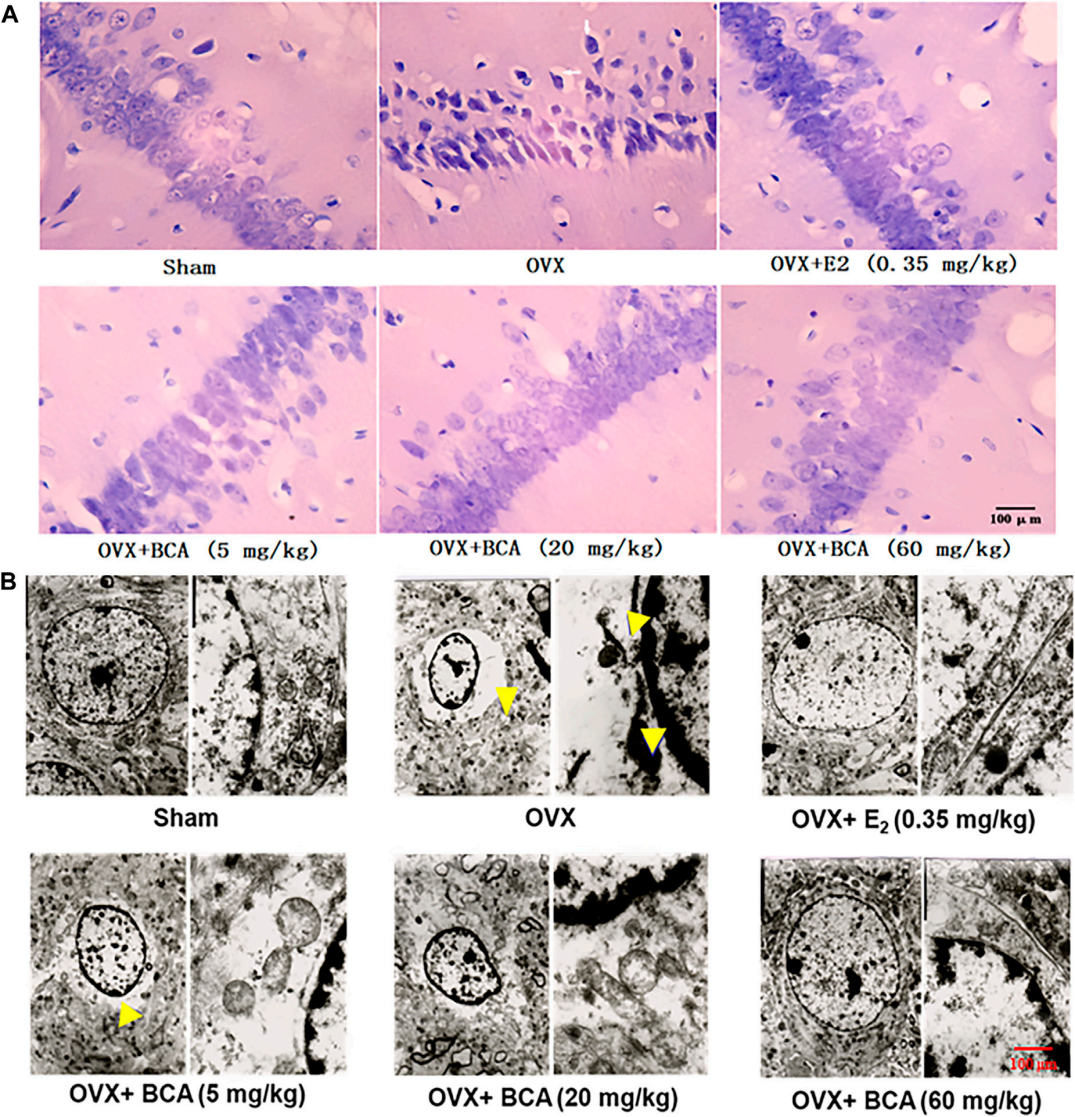
The effects of BCA on morphology and structure of hippocampal neurons in the OVX rats. **(A)** HE staining of hippocampal CA1 subregions. Pyramidal cells in CA1 were extensively damaged, as indicated by reduced cell volume and nuclear chromatin condensation (indicated by yellow arrows) in the OVX rats. **(B)** Ultrastructure changes in hippocampal CA1 *via* electron microscopy. The disappearance of cell membranes, multiple vacuoles around nuclei and nuclear chromatin inside the nuclear membrane were observed in the hippocampus of OVX rats. These abnormal changes in neurons were improved following treatment with E2 or BCA.

In the sham rats, the hippocampal neurons were well arranged with complete cell structure and big and regular nuclei. In the OVX rats, the CA1 neurons were extensively damaged, which was characterized by karyopycnosis and nuclear chromatin condensation (Figure 3A). These abnormalities were markedly attenuated by BCA and estradiol.

To further examine the morphological changes, we observed the hippocampal neurons using electron microscopy. As shown in Figure 3B, neurons in the hippocampus of sham rats showed normal endoplasmic reticulum, mitochondria, and nuclear chromatin. In contrast in the OVX rats, ultrastructural analysis revealed the disappearance of cell membranes, multiple vacuoles around nuclei and nuclear chromatin inside the nuclear membranes of neurons in hippocampus. Administration of BCA or estradiol attenuated the deteriorative vacuolization and aggregation of nuclear chromatin caused by OVX.

### BCA Increased Antioxidant Activity and Reduced Lipid Peroxidation in OVX Rats

**FIGURE 4:**
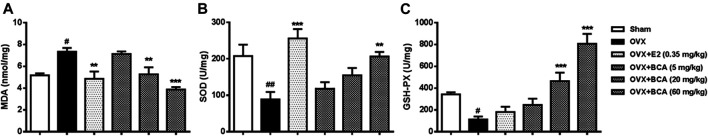
The effects of BCA on MDA **(A)**, SOD **(B)**, and GSH-Px **(C)** in hippocampal tissues of the OVX rats. The contents of MDA and activity of SOD and GSH-Px were measured in hippocampal tissues using ultraviolet spectrophotometry. BCA attenuated oxidative stress in OVX rats. Values shown are means ± S.E. ^#^ *p* < 0.05, ^##^ *p* < 0.01 vs. Sham; **p < 0.01, ***p < 0.001 vs. OVX alone; n = 8.

To investigate the mechanisms of neuroprotective effect of BCA, we measured the levels of MDA, SOD, and GSH-Px in the hippocampus. In the OVX rats, MDA was markedly increased (F _(5,42)_ = 5.47, *p* < 0.05; Figure 4A), while SOD and GSH-Px were decreased (F _(5,42)_ = 12.35, *p* < 0.01 and F _(5,42)_ = 17.26, *p* < 0.05, respectively; Figure 4B,C). These were reversed by estradiol (*p* < 0.01 for MDA and *p* < 0.001 for SOD, but *p* > 0.05 for GSH-Px) and BCA (5–60 mg/kg) in a dose-dependent manner (*p* < 0.01 or *p* < 0.001), suggesting that BCA suppressed brain oxidative stress after estrogen deficiency in OVX rats.

### BCA Inhibited Apoptosis in OVX Rats

**FIGURE 5:**
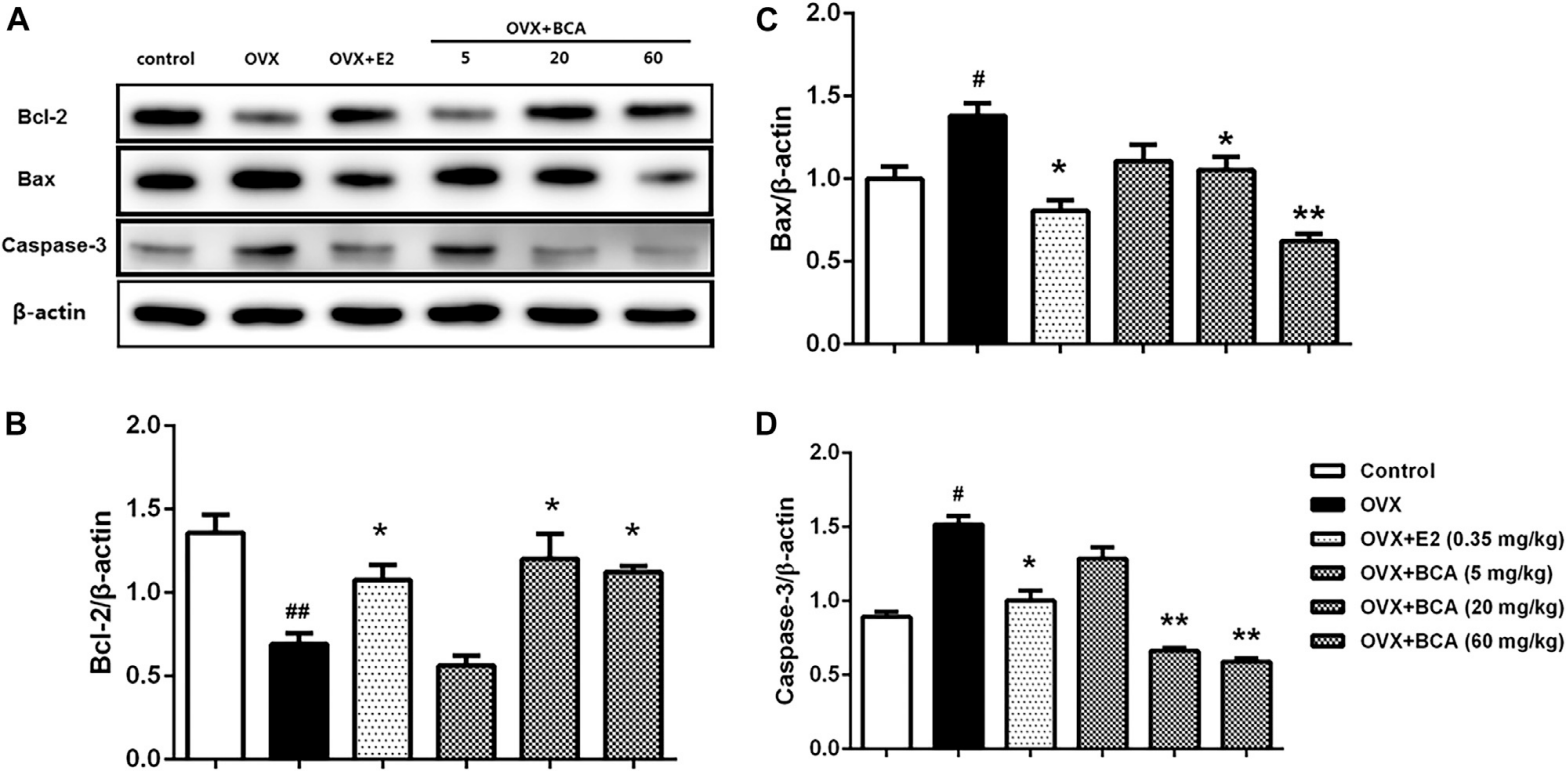
The effects of BCA on apoptosis in the hippocampus of OVX rats. Representative Western blotting and densitometry of Bcl-2, Bax, capase-3, and β-actin. Values shown are means ± S.E. ^#^ *p* < 0.05, ^##^ *p* < 0.01 vs. control; *p < 0.05, **p < 0.01 vs. OVX; n = 3.

To investigate the influence of apoptosis on neuroprotection of BCA, we examined the expression of Bcl-2, Bax, and caspase-3 in the hippocampus of OVX rats. As shown in Figure 5, the expression of Bcl-2 was reduced (F _(5,12)_ = 28.3, *p* < 0.01; Figure 5A,B), whereas the expression of Bax and caspase-3 was increased (F _(5,12)_ = 7.64, *p* < 0.05 and F _(5,12)_ = 8.78, *p* < 0.05, respectively; Figure 5A,C,D) in OVX rats compared to the control. These were reversed by estradiol (*p* < 0.05) and, similarly, by BCA (5–60 mg/kg; *p* < 0.05 or *p* < 0.01), suggesting that BCA inhibited apoptosis in the hippocampus of OVX rats.

### BCA Increased Cell Viability in Primary Cultures of Hippocampal Neurons Treated With H_2_O_2_

**FIGURE 6:**
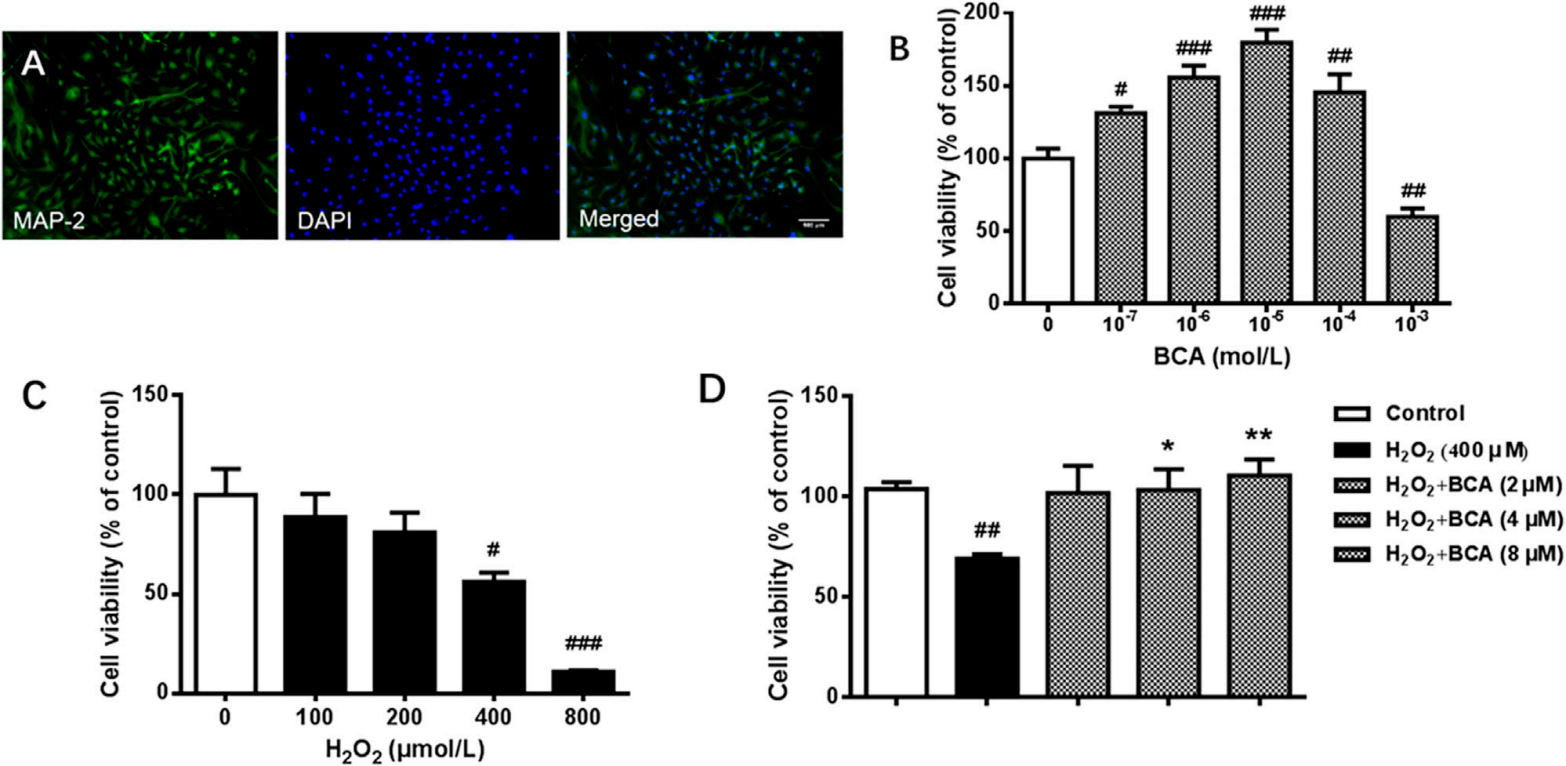
Identification of neurons and determination of cell viability in primary cultures of rat hippocampal neurons. **(A)** Staining of antibodies against MAP-2, a neuron-specific marker, and/or DAPI, a nuclear marker, in hippocampal neurons. A purity of >90% were identified. **(B)** Cell viability in response to 24-h incubation with increasing concentrations (10^−7^ - 10^−3^ mol/L) of BCA. **(C)** Cell vitality in response to 12-h incubation with increasing concentrations of (100–800 µM) of H_2_O_2_. **(D)** Effects of BCA on cell viability in hippocampal neurons treated with H_2_O_2_ (400 µM) for 12 h. BCA was added 24 h before H_2_O_2._ Values shown are means ± S.E. ^#^ *p* < 0.05, ^##^ *p* < 0.01, ^###^ *p* < 0.001 vs. control; *p < 0.05, **p < 0.01 vs. H_2_O_2_ alone; n = 4–6.

To test the effect of BCA on cell viability, we first verified the purification of rat hippocampal neurons by examining the expression of MAP2, a specific marker for neurons. As shown in Figure 6A, most of cells were MAP2 positive, indicating high purity of neurons. Neurons were incubated with different concentrations of BCA for 24 h with or without H_2_O_2_ for additional 12 h before determination of cell viability. As shown in Figure 6B, treatment with BCA at concentrations from 0.1 to 100 µM increased cell viability (F _(5,31)_ = 23.18, *p* < 0.05, *p* < 0.001, *p* < 0.01), indicating neuroprotective properties. At the highest dose of 1,000 μM, BCA produced 40% cell death (*p* < 0.01), indicating neurotoxicity of BCA at high doses. In contrast, H_2_O_2_ at 100–800 µM decreased cell viability in a concentration-dependent manner (F _(4,19)_ = 11.97, *p* < 0.01, *p* < 0.001), with significant cytotoxicity starting at 400 µM (Figure 6C). Therefore, the concentration of 400 µM H_2_O_2_ was used in the following experiments. To further test the protective effects of BCA, neurons were pretreated with BCA for 24 h before exposure to H_2_O_2_ for another 12 h. As shown in Figure 6D, BCA at concentrations of 2, 4, and 8 µM reversed H_2_O_2_-induced decreases in cell viability (F _(4,19)_ = 5.235, *p* < 0.05, *p* < 0.01).

### BCA Mitigated H_2_O_2_-Induced Increases in ROS Levels in Primary Cultures of Hippocampal Neurons

**FIGURE 7:**
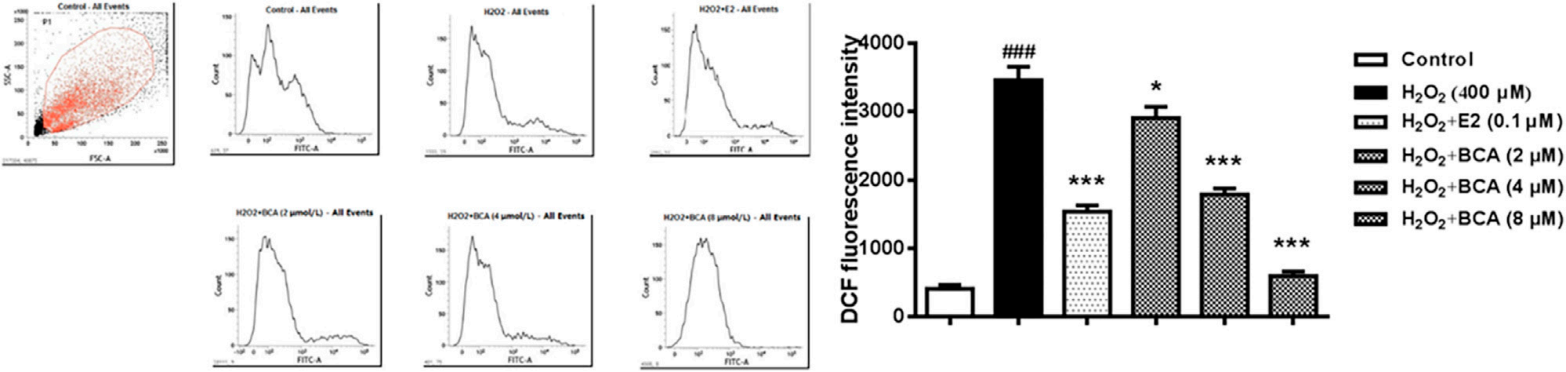
Determination of intracellular ROS levels in primary hippocampal neurons treated with BCA (2–8 µM) or E2 (0.1 µM) in the presence of H_2_O_2_ (400 µM). BCA reduced H_2_O_2_-induced production of ROS, as indicated by DCF fluorescence intensity. Values shown are means ± S.E. ^###^ *p* < 0.001 vs. control; *p < 0.05, ***p < 0.001 vs. H_2_O_2_ alone; n = 3.

To determine whether BCA affected H_2_O_2_-mediated ROS, the production of ROS was examined using flow cytometry in primary cultures of hippocampal neurons. As shown in Figure 7, the ROS production was increased by H_2_O_2_ at 400 µM (*p* < 0.001) compared to the control. This was blocked by BCA (2–8 µM) in a dose-dependent manner (F _(5,12)_ = 53.78, *p* < 0.05, *p* < 0.001), which was similar to E2 (*p* < 0.001), suggesting that BCA alleviated oxidative stress injury.

### BCA Inhibited H_2_O_2_-Induced Apoptosis of Rat Hippocampal Neurons

**FIGURE 8:**
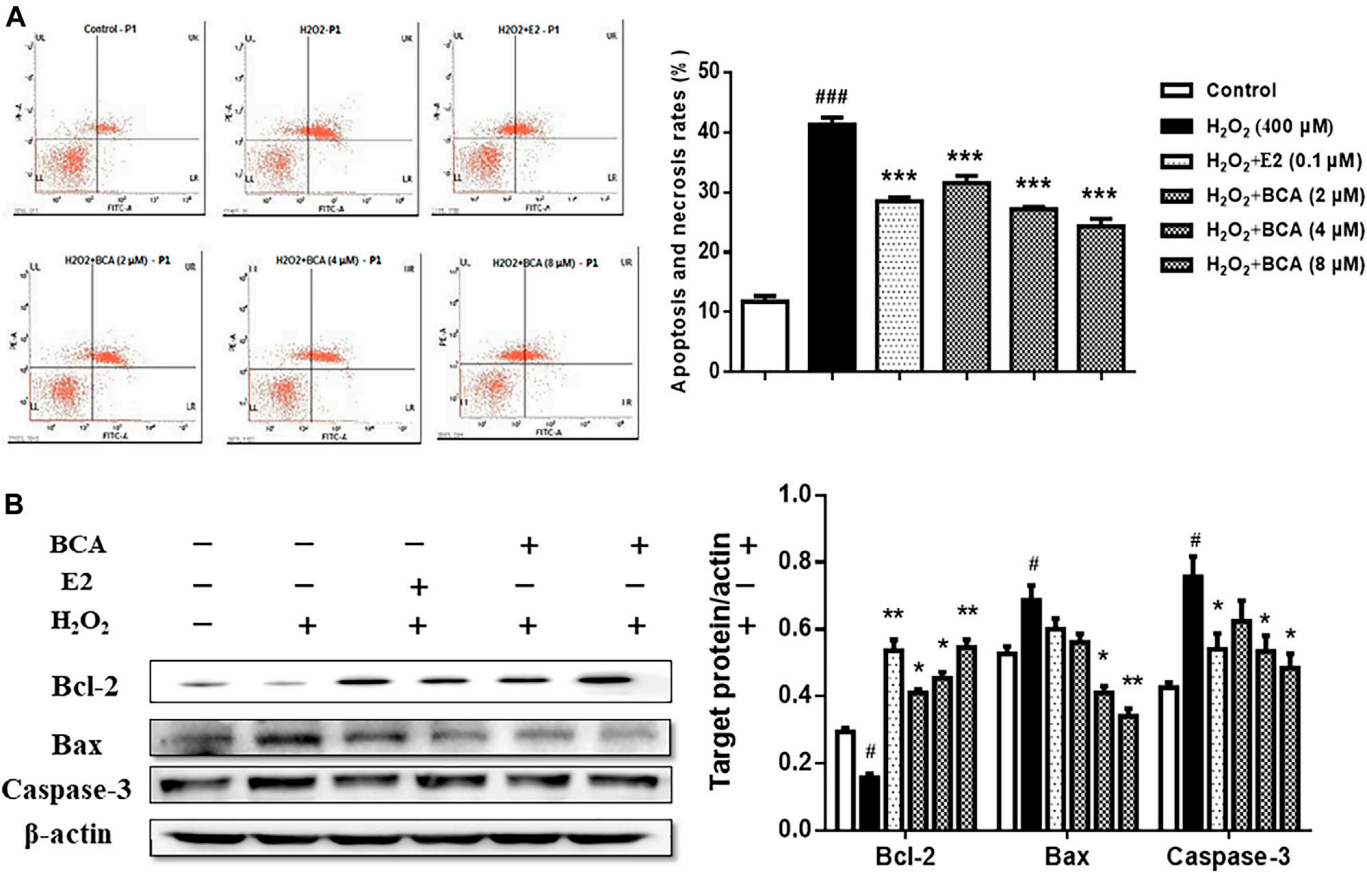
The effects of BCA on apoptosis induced by H_2_O_2_ in primary hippocampal neurons. **(A)** Effects of BCA on the apoptotic rate in the presence of H_2_O_2_ (400 µM) evaluated by flow cytometry. **(B)** Representative Western blotting and densitometry of Bcl-2, Bax, capase-3, and β-actin. Values shown are means ± S.E. ^#^ *p* < 0.05, ^###^ *p* < 0.001 vs. control; *p < 0.05, **p < 0.01, ***p < 0.001 vs. H_2_O_2_ alone; n = 3.

To examine the effect of BCA on apoptosis induced by H_2_O_2_, we examined the apoptotic rate and the expression of Bcl-2, Bax, and caspase-3 in primary cultures of hippocampal neurons treated with BCA in the presence of H_2_O_2_. As shown in Figure 8A, H_2_O_2_-induced increase (*p* < 0.001 vs. control) in apoptotic rate was suppressed by BCA (2–8 μM; F _(5,12)_ = 16.37, *p* < 0.001) in a dose-dependent manner, similar to E2 (0.1 µM). The expression of Bcl-2 was reduced (F _(5,12)_ = 6.86, *p* < 0.05), whereas the expression of Bax and caspase-3 was increased (Bax, F _(5,12)_ = 5.33, *p* = 0.01; caspase-3, F _(5,12)_ = 4.32, *p* < 0.05) by H_2_O_2_ compared to the control. These were reversed by estradiol (*p* < 0.01 for Bcl-2 and *p* < 0.05 for caspase-3) and, similarly, by BCA (2–8 μM; *p* < 0.05, *p* < 0.01) in a dose-dependent manner (Figure 8B), suggesting that BCA inhibited H_2_O_2_-induced apoptosis in hippocampal neurons.

## Discussion

Recent translational brain imaging studies have demonstrated that women at 40–60 years old exhibit an AD-endophenotype characterized by decreased metabolic activity, including reduced glucose metabolism and mitochondrial function, and increased brain deposition of amyloid-β (Aβ), a hallmark of AD pathology, as compared to age-matched men (Mosconi et al., 2017). Gender difference may arise from the fact that women are characterized by a relatively abrupt estradiol drop and men by a steady decrease of testosterone (Barron and Pike, 2012). Thus, sex hormones are likely modulators of AD susceptibility (Keyvani et al., 2018). Nonetheless, of all gonadal hormones, estrogen appears to be particularly involved in the pathophysiology of AD-dementia in women. Estrogen plays a protective role in AD and dementia; estrogen dysfunction seems to exacerbate or perhaps precipitate the AD process in women (Rahman et al., 2019). Here in the present study, we provided additional demonstration that estrogen deficiency impaired learning and memory and disrupted the morphological function of neurons in female rats. These were attenuated by treatment with BCA, a natural O-methylated isoflavonoid phytoestrogen derived from red clover or chickpea.

Ovariectomized animal models have been widely used to study diseases associated with decreased estradiol secretion. Indeed, the use of OVX rats or mice to mimic the postmenopausal conditions in women is well established and represents a reliable and reproducible model (Chalvon-Demersay et al., 2017). OVX produced increases in body weights, which is considered to be related to increases in the visceral and subcutaneous fat mass and reduction of the metabolic rate, as observed in OVX mice (Guillerminet et al., 2012). However, in mice lacking estrogen receptor (ER) α in the central nervous system, hyperphagia and decreased energy expenditure have also been reported (Xu et al., 2019). This is consistent with the results in our study that BCA and E2 prevented increases in body weights and fat mass induced by OVX in female rats.

Estrogen affects various brain regions, leading to influences on cognitive function, affect, and behavior (Fink et al., 1996; Brinton et al., 2015). Estrogen produces neuroprotective properties through its effects on spinogenesis, protecting the brain from age-related, toxic insults (Gould et al., 1990; Woolley and McEwen, 1992). It also protects DNA against damage induced by H_2_O_2_ and arachidonic acid (Rettberg et al., 2014). Further, estrogen is fundamental in the metabolic regulation of the brain and body (Brinton et al., 2015). It regulates glucose transport, aerobic glycolysis, and mitochondrial function to generate ATP in the brain (Woolley and McEwen, 1992).

Since HRT is used to treat common symptoms of menopause, it may also reduce the risk of AD. However, the use of HRT for treating AD has been challenged by its side effects such as blood clots, heart attacks, strokes, breast cancer, and gall bladder disease, especially at high doses and for a long-term use (Menopause and Hormones, 2004). It is important to develop novel, effective treatments with minimum side effects. Many recent studies have shown that phytoestrogens produce neuroprotective effects in animal models of AD (Soni et al., 2014; Hussain et al., 2018). Phytoestrogens have a structural similarity to 17β-estradiol and selectively binds to ERs, leading to the regulation of expression of related genes and producing estrogenic or antiestrogenic effects (Lecomte et al., 2017). It has been shown that BCA exhibits broad pharmacological functions such as anti-tumor (Youssef et al., 2016), anti-oxidation (Mishra et al., 2008), and antihyperglycemic activity (Harini et al., 2012) with less side effects (Schrepfer et al., 2006; Reiter et al., 2011). In the present study, we demonstrated that, similar to estradiol, BCA markedly enhanced learning and memory in OVX rats and improved the structure and degenerative changes in the hippocampal neurons. These are supported by studies elsewhere showing that BCA alleviates vascular dysfunction and dementia in dyslipidemic ovariectomized rats (Verma and Sharma, 2015) and reverses memory deficits induced by scopolamine or aging in mice (Biradar et al., 2014).

It has been well documented that estrogen depletion produces oxidative stress (Désiré et al., 2015). Consistent with this, female rats with ovariectomy displayed increased MDA and decreased GSH and GSH-Px in the hippocampus of the brain, indicating increased oxidative stress. BCA mimicked the ability of E2 to attenuate OVX-induced oxidative stress in rats. This was supported by the *in vitro* experiment in primary cultures of hippocampal neurons, in which BCA reversed H_2_O_2_-induced increases in ROS, a critical factor causing oxidative stress and cell damage (Yürüker et al., 2015; Selli et al., 2016). More specifically for the latter, H_2_O_2_-induced cell viability was reversed by BCA treatment, which produced anti-apoptotic activity, as demonstrated both in the hippocampus *in vivo* and in primary cultures of hippocampal neurons *in vitro*. This is in agreement with the finding that BCA attenuates Aβ_25–35_-induced PC12 cell injury and apoptosis (Tan and Kim, 2016). In addition, it has been confirmed that BCA reduces inflammatory injury and neuronal apoptosis following subarachnoid hemorrhage and cerebral ischemia/reperfusion injury (Wu et al., 2018; Guo et al., 2019). Together, these results suggest that BCA produces neuroprotection likely *via* its anti-oxidative and anti-apoptotic properties.

It was noted that E2 did not produce statistically significant blocking effects on OVX- or H_2_O_2_-induced changes in some measures, including the time spent in the target quadrant in the MWM and levels of GSH-Px in the brain of OVX rats, and expression of Bax in H_2_O_2_-treated neurons. These variations might be due to the dose or concentration of E2 selected in the study. A higher dose/concentration of E2 may be needed in future studies. Regardless of this, the observations did not alter the conclusion because E2 was effective in other related measures at the dose/concentration tested and, more importantly, BCA was significantly effective in all the tests or assays either *in vivo* or *in vitro*.

## Conclusion

Ovariectomy produces deleterious effects on neurons in the brain. It impairs cognitive ability and produces oxidative stress and neuron apoptosis. The present study provides promising demonstration that BCA reverses OVX-induced cognition impairment and neuron damage in the hippocampus of the brain. It also attenuates H_2_O_2_-induced oxidation and apoptosis in the hippocampus and/or hippocampal neurons. Overall, the results suggest that BCA enhances learning and memory *via* neuroprotection mediated by its antioxidant and anti-apoptotic properties under the circumstance of estrogen deficiency. BCA can be used for treatment of brain dysfunctions such as dementia in postmenopausal women.

## Data Availability

The raw data supporting the conclusions of this article will be made available by the authors, without undue reservation.

## References

[B1] Aguiar R. B. Dickel O. E. Cunha R. W. Monserrat J. M. Barros D. M. Martinez P. E. (2006). Estradiol valerate and tibolone: effects on memory. Pharmacol. Biochem. Behav. 85 (4), 689–696. 10.1016/j.pbb.2006.10.023 17169418

[B2] Alauddin Chaturvedi. S. Chaturvedi S. Malik M. Y. Azmi L. Shukla I. Naseem Z. (2018). Formononetin and biochanin A protects against ritonavir induced hepatotoxicity *via* modulation of NfκB/pAkt signaling molecules. Life Sci. 213, 174–182. 10.1016/j.lfs.2018.10.023 30326221

[B3] Banker G. A. Cowan W. M. (1977). Rat hippocampal neurons in dispersed cell culture. Brain Res. 126 (3), 397–442. 10.1016/0006-8993(77)90594-7 861729

[B4] Barron A. M. Pike C. J. (2012). Sex hormones, aging, and Alzheimer’s disease, Front. Biosci.(Elite Ed) 4, 976–997. 22201929 10.2741/e434 PMC3511049

[B5] Beral V. Million Women Study Collaborators (2003). Breast cancer and hormone-replacement therapy in the million women study. Lancet 362 (9382), 419–427. 10.1016/s0140-6736(03)14065-2 12927427

[B6] Best T. M. Fiebig R. Corr D. T. Brickson S. Ji L. (1999). Free radical activity, antioxidant enzyme, and glutathione changes with muscle stretch injury in rabbits. J. Appl. Physiol. 87 (1), 74–82. 10.1152/jappl.1999.87.1.74 10409559

[B7] Biradar S. M. Joshi H. Chheda T. K. (2014). Biochanin-A ameliorates behavioural and neurochemical derangements in cognitive-deficit mice for the betterment of Alzheimer’s disease. Hum. Exp. Toxicol. 33 (4), 369–382. 10.1177/0960327113497772 23900307

[B8] Brinton R. D. Yao J. Yin F. Mack W. J. Cadenas E. (2015). Perimenopause as a neurological transition state. Nat. Rev. Endocrinol. 11 (7), 393–405. 10.1038/nrendo.2015.82 26007613 PMC9934205

[B9] Brookmeyer R. Evans D. A. Hebert L. Langa K. M. Heeringa S. G. Plassman B. L. (2011). National estimates of the prevalence of Alzheimer’s disease in the United States. Alzheimers Dement. 7, 61–73. 10.1016/j.jalz.2010.11.007 21255744 PMC3052294

[B10] Carroll J. C. Rosario E. R. Chang L. Stanczyk F. Z. Oddo S. LaFerla F. M. (2007). Progesterone and estrogen regulate Alzheimer-like neuropathology in female 3xTg-AD mice. J. Neurosci. 27 (48), 13357–13365. 10.1523/JNEUROSCI.2718-07.2007 18045930 PMC6673397

[B11] Chalvon-Demersay T. Blachier F. Tomé D. Blais A. (2017). Animal models for the study of the relationships between diet and obesity: a focus on dietary protein and estrogen deficiency. Front. Nutr. 4 (4), 5. 10.3389/fnut.2017.00005 28373974 PMC5357654

[B12] Chapman R. M. Mapstone M. Gardner M. N. Sandoval T. C. McCrary J. W. Guillily M. D. (2011). Women have farther to fall: gender differences between normal elderly and Alzheimer's disease in verbal memory engender better detection of Alzheimer’s disease in women. J. Int. Neuropsychol. Soc. 17 (4), 654–662. 10.1017/S1355617711000452 21486518 PMC3387297

[B13] Chen H. Q. Jin Z. Y. Li G. H. (2007). Biochanin A protects dopaminergic neurons against lipopolysaccharide-induced damage through inhibition of microglia activation and proinflammatory factors generation. Neurosci. Lett. 417 (2), 112–117. 10.1016/j.neulet.2006.11.045 17399896

[B14] Désiré D. D. P. Yolande Sandrine M. N. Danielle Claude B. Mireille K. Oumarou Bibi-Farouck A. Théophile D. (2015). *In vivo* estrogenic-like activities of Gouania longipetala Hemsl. (Rhamnaceae) bark extracts in a post-menopause-like model of ovariectomized Wistar rats. J. Ethnopharmacol. 168, 122–128. 10.1016/j.jep.2015.03.049 25849733

[B15] Engler-Chiurazzi E. B. Singh M. Simpkins J. W. (2016). Reprint of: from the 90’s to now: a brief historical perspective on more than two decades of estrogen neuroprotection. Brain Res. 1645, 79–82. 10.1016/j.brainres.2016.06.016 27317847 PMC4969093

[B16] Facecchia K. Fochesato L. A. Ray S. D. Stohs S. J. Pandey S. (2011). Oxidative toxicity in neurodegenerative diseases: role of mitochondrial dysfunction and therapeutic strategies. J. Toxicol. 2011, 683728. 10.1155/2011/683728 21785590 PMC3139184

[B17] Fink G. Sumner B. E. Rosie R. Grace O. Quinn J. P. (1996). Estrogen control of central neurotransmission: effect on mood, mental state, and memory. Cell. Mol. Neurobiol. 16 (3), 325–344. 10.1007/BF02088099 8818400 PMC11563142

[B18] Gao M. Ji S. Li J. Zhang S. (2019). DL-3-n-butylphthalide (NBP) ameliorates cognitive deficits and CaMKII-mediated long-term potentiation impairment in the hippocampus of diabetic db/db mice. Neurol. Res. 41 (11), 1024–1033. 10.1080/01616412.2019.1672387 31578943

[B19] Gould E. Woolley C. S. Frankfurt M. McEwen B. S. (1990). Gonadal steroids regulate dendritic spine density in hippocampal pyramidal cells in adulthood. J. Neurosci. 10 (4), 1286–1291. 10.1523/jneurosci.10-04-01286.1990 2329377 PMC6570209

[B20] Guillerminet F. Fabien-Soulé V. Even P. C. Tomé D. Benhamou C. L. Roux C. (2012). Hydrolyzed collagen improves bone status and prevents bone loss in ovariectomized C3H/HeN mice. Osteoporos. Int. 23, 1909–1919. 10.1007/s00198-011-1788-6 21927918

[B21] Guo M. Lu H. Qin J. Qu S. Wang W. Guo Y. (2019). Biochanin A provides neuroprotection against cerebral ischemia/reperfusion injury by nrf2-mediated inhibition of oxidative stress and inflammation signaling pathway in rats. Med. Sci. Mon. Int. Med. J. Exp. Clin. Res. 25, 8975–8983. 10.12659/msm.918665 PMC6896748 31767824

[B22] Harini R. Ezhumalai M. Pugalendi K. V. (2012). Antihyperglycemic effect of biochanin A, a soy isoflavone, on streptozotocin-diabetic rats. Eur. J. Pharmacol. 676 (1–3), 89–94. 10.1016/j.ejphar.2011.11.051 22178203

[B23] Henderson V. W. Brinton R. D. (2010). Menopause and mitochondria: windows into estrogen effects on Alzheimer’s disease risk and therapy. Prog. Brain Res. 182, 77–96. 10.1016/S0079-6123(10)82003-5 20541661 PMC5776041

[B24] Hussain A. Tabrez E. S. Muhammad A. Peela J. R. (2018). The mechanisms of dietary phytoestrogen as a potential treatment and prevention agent against alzheimer’s disease. Crit. Rev. Eukaryot. Gene Expr. 28 (4), 321–327. 10.1615/critreveukaryotgeneexpr.2018025847 30311580

[B25] Keyvani K. Münster Y. Kurapati N. K. Rubach S. Schönborn A. Kocakavuk E. (2018). Higher levels of kallikrein-8 in female brain may increase the risk for Alzheimer’s disease. Brain Pathol. 28 (6), 947–964. 10.1111/bpa.12599 29505099 PMC8028555

[B26] Lecomte S. Demay F. Ferrière F. Pakdel F. (2017). Phytochemicals targeting estrogen receptors: beneficial rather than adverse effects?. Int. J. Mol. Sci. 18 (7), 1381. 10.3390/ijms18071381 PMC5535874 28657580

[B27] Liu S. B. Han J. Zhang N. Tian Z. Li X. B. Zhao M. G. (2011). Neuroprotective effects of oestrogen against oxidative toxicity through activation of G-protein-coupled receptor 30 receptor. Clin. Exp. Pharmacol. Physiol. 38 (9), 577–585. 10.1111/j.1440-1681.2011.05549.x 21645039

[B28] Menopause and Hormones (2004). “what can you believe”. AWHONN Lifelines 8 (6), 493–494. 15690751

[B50] Michael C. (2016). Why we need research about autism and ageing. Autism, 20 (5), 515–516. 10.1177/1362361316647224 27141078

[B29] Mishra P. Kale R. K. Kar A. (2008). Chemoprevention of mammary tumorigenesis and chemomodulation of the antioxidative enzymes and peroxidative damage in prepubertal Sprague Dawley rats by Biochanin A. Mol. Cell. Biochem. 312 (1-2), 1–9. 10.1007/s11010-008-9714-8 18273562

[B30] Mosconi L. Berti V. Guyara-Quinn C. McHugh P. Petrongolo G. Osorio R. S. (2017). Perimenopause and emergence of an Alzheimer’s bioenergetic phenotype in brain and periphery. PloS One 12 (10), e0185926. 10.1371/journal.pone.0185926 29016679 PMC5634623

[B31] Pike C. J. Carroll J. C. Rosario E. R. Barron A. M. (2009). Protective actions of sex steroid hormones in Alzheimer’s disease. Front. Neuroendocrinol. 30 (2), 239–258. 10.1016/j.yfrne.2009.04.015 19427328 PMC2728624

[B32] Rahman A. Jackson H. Hristov H. Isaacson R. S. Saif N. Shetty T. (2019). Sex and gender driven modifiers of alzheimer's: the role for estrogenic control across age, race, medical, and lifestyle risks. Front. Aging Neurosci. 11, 315. 10.3389/fnagi.2019.00315 31803046 PMC6872493

[B33] Reiter E. Gerster P. Jungbauer A. (2011). Red clover and soy isoflavones--an *in vitro* safety assessment. Gynecol. Endocrinol. 27 (12), 1037–1042. 10.3109/09513590.2011.588743 21801124

[B34] Rettberg J. R. Yao J. Brinton R. D. (2014). Estrogen: a master regulator of bioenergetic systems in the brain and body. Front. Neuroendocrinol. 35 (1), 8–30. 10.1016/j.yfrne.2013.08.001 23994581 PMC4024050

[B35] Rocca W. A. Bower J. H. Maraganore D. M. Ahlskog J. E. Grossardt B. R. de Andrade M. (2008). Increased risk of parkinsonism in women who underwent oophorectomy before menopause. Neurology 70 (3), 200–209. 10.1212/01.wnl.0000280573.30975.6a 17761549

[B36] Rocca W. A. Grossardt B. R. Shuster L. T. (2010). Oophorectomy, menopause, estrogen, and cognitive aging: the timing hypothesis. Neurodegener. Dis. 7 (1-3), 163–166. 10.1159/000289229 20197698 PMC2859235

[B37] Schrepfer S. Deuse T. Münzel T. Schäfer H. Braendle W. Reichenspurner H. (2006). The selective estrogen receptor-beta agonist biochanin A shows vasculoprotective effects without uterotrophic activity. Menopause 13 (3), 489–499. 10.1097/01.gme.0000185941.63497.10 16735947

[B38] Selli J. Unal D. Mercantepe F. Akaras N. Kabayel R. Unal B. (2016). Protective effects of beta glucan in brain tissues of post-menopausal rats: a histochemical and ultra-structural study. Gynecol. Endocrinol. 32 (3), 234–239. 10.3109/09513590.2015.1110139 26486170

[B39] Sklenickova O. Flesar J. Kokoska L. Vlkova E. Halamova K. Malik J. (2010). Selective growth inhibitory effect of biochanin A against intestinal tract colonizing bacteria. Molecules 15 (3), 1270–1279. 10.3390/molecules15031270 20335979 PMC6257273

[B40] Soni M. Rahardjo T. B. Soekardi R. Sulistyowati Y. Lestariningsih A. Yesufu-Udechuku A. (2014). Phytoestrogens and cognitive function: a review. Maturitas 77 (3), 209–220. 10.1016/j.maturitas.2013.12.010 24486046

[B41] Tan J. W. Kim M. K. (2016). Neuroprotective effects of biochanin A against β-amyloid-induced neurotoxicity in PC12 cells *via* a mitochondrial-dependent apoptosis pathway. Molecules 21 (5), 548. 10.3390/molecules21050548 PMC6274559 27120593

[B42] Verma A. Sharma S. (2015). Beneficial effect of protein tyrosine phosphatase inhibitor and phytoestrogen in dyslipidemia-induced vascular dementia in ovariectomized rats. J. Stroke Cerebrovasc. Dis. 24 (11), 2434–2446. 10.1016/j.jstrokecerebrovasdis.2015.02.019 26324516

[B43] Woolley C. S. McEwen B. S. (1992). Estradiol mediates fluctuation in hippocampal synapse density during the estrous cycle in the adult rat. J. Neurosci. 12 (7), 2549–2554. 10.1523/jneurosci.12-07-02549.1992 1613547 PMC6575846

[B44] Wu L. Y. Ye Z. N. Zhuang Z. Gao Y. Tang C. Zhou C. H. (2018). Biochanin a reduces Inflammatory injury and neuronal apoptosis following subarachnoid hemorrhage via suppression of the TLRs/TIRAP/MyD88/NF-kappaB pathway. Behav. Neurol., 2018 (3), 1960106. 10.1155/2018/1960106 29971136 PMC6008698

[B45] Xu Y. Nedungadi T. P. Zhu L. Sobhani N. Irani B. G. Davis K. E. (2019). Distinct hypothalamic neurons mediate estrogenic effects on energy homeostasis and reproduction. Cell Metabol. 29 (5), 1232. 10.1016/j.cmet.2019.04.006 PMC6553462 31067449

[B46] Youssef M. M. Tolba M. F. Badawy N. N. Liu A. W. El-Ahwany E. Khalifa A. E. (2016). Novel combination of sorafenib and biochanin-A synergistically enhances the anti-proliferative and pro-apoptotic effects on hepatocellular carcinoma cells. Sci Rep. 6, 30717. 10.1038/srep30717 27470322 PMC4965826

[B47] Yürüker V. Nazıroğlu M. Şenol N. (2015). Reduction in traumatic brain injury-induced oxidative stress, apoptosis, and calcium entry in rat hippocampus by melatonin: possible involvement of TRPM2 channels. Metab. Brain Dis. 30 (1), 223–231. 10.1007/s11011-014-9623-3 25339252

[B48] Zhang H. T. Huang Y. Masood A. Stolinski L. R. Li Y. Zhang L. (2008). Anxiogenic-like behavioral phenotype of mice deficient in phosphodiesterase 4B (PDE4B). Neuropsychopharmacology 33, 1611–1623. 10.1038/sj.npp.1301537 17700644 PMC2728355

[B49] Zhou Q. Zhu L. Zhang D. Li N. Li Q. Dai P. (2016). Oxidative stress-related biomarkers in postmenopausal osteoporosis: a systematic review and meta-analyses. Dis. Markers 2016, 7067984. 10.1155/2016/7067984 27594735 PMC4995322

